# A retrospective analysis of setup and intrafraction positional variation in stereotactic radiotherapy treatments

**DOI:** 10.1002/acm2.13076

**Published:** 2020-11-03

**Authors:** Micah Barnes, Adam Yeo, Kenton Thompson, Claire Phillips, Tomas Kron, Nicholas Hardcastle

**Affiliations:** ^1^ Department of Physical Sciences Peter MacCallum Cancer Centre Melbourne VIC Australia; ^2^ Imaging and Medical Beamline Australian Nuclear Science and Technology Organisation ‐ Australian Synchrotron Clayton VIC Australia; ^3^ School of Science RMIT University Melbourne VIC Australia; ^4^ Department of Radiation Therapy Services Peter MacCallum Cancer Centre Melbourne VIC Australia; ^5^ Department of Radiation Oncology Peter MacCallum Cancer Centre Melbourne VIC Australia; ^6^ Sir Peter MacCallum Department of Oncology The University of Melbourne Melbourne VIC Australia; ^7^ Centre for Medical Radiation Physics University of Wollongong Wollongong NSW Australia

**Keywords:** ExacTrac, IGRT, image guidance, intrafraction motion, patient position, SRS, SRT, thermoplastic mask

## Abstract

**Purpose:**

The aim of this study was to provide a comprehensive assessment of patient intrafraction motion in linac‐based frameless stereotactic radiosurgery (SRS) and radiotherapy (SRT).

**Methods:**

A retrospective review was performed on 101 intracranial SRS/SRT patients immobilized with the Klarity stereotactic thermoplastic mask (compatible with the Brainlab frameless stereotactic system) and aligned on a 6 Degree of Freedom (DoF) couch with the Brainlab ExacTrac image guidance system. Both pretreatment and intrafraction correction data are provided as observed by the ExacTrac system. The effects of couch angle and treatment duration on positioning outcomes are also explored.

**Results:**

Initial setup data for patients is shown to vary by up to ±4.18 mm, ±2.97°, but when corrected with a single x‐ray image set with ExacTrac, patient positions are corrected to within ±2.11 mm, ±2.27°. Intrafraction patient motion is shown to be uniformly random and independent of both time and couch angle. Patient motion was also limited to within approximately 3 mm, 3° by the thermoplastic mask.

**Conclusions:**

Our results indicate that since patient intrafraction motion is unrelated to couch rotation and treatment duration, intrafraction patient monitoring in 6 DoF is required to minimize intracranial SRS/SRT margins.

## 1 INTRODUCTION

Stereotactic radiosurgery (SRS) has long been the preferred technique for treating intracranial metastases due to its ability to achieve ablative doses with high conformity and spatial accuracy to small targets. Due to the potential for complications such as brain necrosis with high doses employed in SRS,^1^ small margins are required to limit the irradiated volume of surrounding organs at risk. As a result, the small margins employed by SRS require both accurate patient alignment to within sub‐millimeter precision and patient immobilization or monitoring. Recently, the use of frameless thermoplastic face masks are preferred over the traditional stereotactic frames for patient immobilization due to their noninvasive nature and effective restriction of patient motion.^2^ The use of frameless immobilization necessitates image guidance, since the patient is now no longer indexed to the treatment delivery system.

Approaches for image guidance for SRS can vary between institutions. Typically, alignment to skull bone structures is performed due to the assumed rigid relationship between brain targets and bone anatomy.^3^ In linac‐based SRS, cone beam CT is often used for visualization of intracranial structures, however, it has limitations at nonzero couch angles. Planar orthogonal imaging at the linac (kV‐kV or kV‐MV) is also commonly used as it can be acquired from all couch angles, such as with dedicated SRS IGRT systems. One such dedicated IGRT system is the Brainlab ExacTrac Frameless SRS system (Brainlab AG, Germany), which utilizes orthogonal floor‐mounted kV tubes and ceiling mounted x‐ray detectors.

Over the past decade, several studies have been published investigating patient position stability in stereotactic radiotherapy using stereoscopic, linac‐mounted planar or volumetric imaging. Several studies have investigated inter‐ and intra‐fraction patient movement through analysis of pre‐ and/or post‐treatment imaging.^2^, ^4^, ^5^, ^6^, ^7^, ^8^ Further, other studies have provided more granular intrafraction motion data through collection of x‐ray images at a range of frequencies during treatment.^9^, ^10^, ^11^, ^12^, ^13^ Many however are limited to small patient cohorts or provide only 3 Degrees of Freedom (DoF) or 4 DoF information, or other limited subsets unique to the aims of their experiment. Thus, there are limited data describing the complete picture of patient motion during treatment.

Several opposing findings exist amongst these studies. For example, Badakhshi et al. (2013) found that increased intrafraction imaging frequency is necessary for small margins on account of patient movement^12^ and Tarnavski et al. (2016) further added that patient positioning errors become larger with respect to treatment time.^14^ Conversely, Lewis et al. (2018) concluded that intrafraction imaging could be reduced after observing minimal patient motion during treatment.^15^ In addition, one study found that the type of immobilization masks used in intracranial SRS have a large effect on positional outcomes,^16^ while another found that in some cases, no differences were observed.^7^

In this study, we perform an offline review on a complete dataset that covers both initial setup and intrafraction motion over a wide range of couch angles in 6 DoF of intracranial SRS and stereotactic radiotherapy (SRT) patients. Our dataset and analysis will enable well‐informed further studies in treatment planning and delivery techniques and potential margins. We hypothesize that, due to patient motion, intrafraction image guidance frequency cannot be reduced in order to keep intracranial SRT/SRS margins small.

## 2 MATERIALS AND METHODS

### 2.A Patient Cohort

For this retrospective review we extracted patient setup data from the Brainlab ExacTrac system. Our inclusion criteria was any patient receiving intracranial SRS/SRT on a Varian TrueBeam STx, equipped with the Brainlab ExacTrac image guidance system (v6.2) and Brainlab 6D couch‐top, immobilized using the Klarity thermoplastic masks (Klarity Medical Products, OH, USA) in conjunction with the Brainlab frameless stereotactic fixation system. These patients included those treated for intact metastases or surgical cavities. Due to data accessibility difficulties, only data from the last year were included in this study.

Between June 2018 to May 2019, 319 patients received SRS/SRT at our institution. Of these, 103 (32%) were identified as suitable for this study based on treatment sites and fractionation regimes. Two patients were further excluded from this study due to the use of different immobilization systems, leaving a total of 101 patients for the analysis. The intact metastases cohort contained 87 patients, with 39 patients treated in a single fraction, and 48 in 2–5 fractions (3.4 ± 1.0, *µ ± σ*). Fifty‐eight patients presented a single metastasis, while the remaining 29 had between 2 and 5 metastases (2.6 ± 0.8). The cavity cohort contained 16 patients, with one patient treated in a single fraction, and 15 in 2–5 fractions (3.6 ± 0.9). Fourteen patients each had a single cavity, whilst the remaining two patients had two and three cavities. For this analysis, the intact metastases and postsurgical cavity patient cohorts were combined, giving a total of 258 treatment fractions, in which each fraction represents a treatment with a set of patient positioning data recorded by ExacTrac.

### 2.B Treatment planning and workflow

Treatments were planned in Brainlab iPlan (v4.5) (Brainlab AG, Germany) using 1 mm CT slice thicknesses. One isocenter per target volume was prescribed, with 1 mm CTV‐PTV margin expansions for intact metastases patients and 2 mm for cavity patients. A dynamic conformal arc (DCAT) technique was used for the majority of cases. Cases with more complex geometry and within close proximity of organs at risk were planned with intensity modulated radiation therapy (IMRT). For the treatments in this patient cohort, our institution defines action levels for patient positioning with ExacTrac as 0.7 mm, 0.7°. When the patient position is below these values in all 6 DoF, the patient is considered to be aligned; if any translation or rotation is above these values then a correctional shift is applied. The general image guidance workflow employed during treatment is described in three steps (grouped by treatment workflow).

(1a) Initial patient setup (with non‐ionizing radiation). First, with the couch at 0° (C0) and the gantry at 0°, the patients were positioned on the couch and immobilized in the stereotactic mask. The radiation therapist then aligned the patients to the visible in‐room lasers, coarsely aligning the target volume to the linac isocenter to within several millimeters. The ExacTrac infrared (IR) system was then used in conjunction with reflective IR markers on the stereotactic mask frame to refine the frame alignment, in 3 DOF, to within 0.7 mm. The alignment of the treatment isocenter to the linac isocenter is now expected to be within 2 mm.

(1b) Initial alignment (with x‐rays). With the couch and gantry still at 0°, the first x‐ray image set was taken with ExacTrac, the outcome of which describes the accuracy of the initial patient alignment in step (1a). ExacTrac then provided a 6 DoF shift to align the patient to the planned position; this shift, if above the action level, was applied without verification.

(2) First planned couch angle. The couch was rotated to the first planned couch position. The patient was then repeatedly imaged and repositioned until it was verified that all 6 DoF were within the action level. Once the patient position was verified, the first arc was delivered.

(3) Intrafraction image guidance. After delivery of the first arc, the patient received image guided alignment at each planned couch angle. For each alignment, ExacTrac was used to obtain and apply 6 DoF shifts until all 6 DoF were within tolerance.

The generalized treatment workflow was as follows: first, treat with the couch at 0° (C0), and step through from C90 to C270 (anti‐clockwise) as planned. This workflow, however, was not always strictly followed. In some cases, the first planned couch was nonzero due to either (a) the absence of a planned arc at C0 or (b) iPlan opted for delivering the C0 arc after delivering other arcs first.

During initial patient setup, or during treatment, if the treating staff determined that the verification shifts calculated by ExacTrac were too large (in excess of 5 mm or 2°), or that the patient position was not a suitable match to the planned position, the patient setup was repeated. The mask was removed and refitted, and the setup procedure restarted. If this procedure occurred mid‐treatment, the patient was then immediately returned to the previous couch angle at which their alignment was invalidated.

Given the treatment workflow, our analysis of patient motion is divided into two accompanying components. Firstly, we review patient setup and verification shifts (covered in 1a, 1b and 2) and secondly, we review patient intrafraction motion (covered in 3). Finally, we further investigate the effects of treatment times on patient positioning.

### 2.C Data analysis

Patient alignment data were collected via the ExacTrac *Export Summary* functionality, which provides the ExacTrac outputs in a comma separated values (CSV) file for each patient treatment plan. The ExacTrac summary report only provides a date for each alignment, therefore, timestamps for the alignments were extracted from the DICOM header of the saved ExacTrac images, and matched to the alignments in the ExacTrac summary file. The information in the csv files, as well as the patient imaging data, was collated into an SQL database for review (SQLite v3.27). Patient translations are defined in the lateral, longitudinal and vertical directions and rotations are defined in the lateral (pitch), longitudinal (roll) and vertical (yaw) directions.

Statistical analysis was performed on the dataset using Python v3.7.2 in conjunction with the NumPy package (v1.17.2) and SciPy (v1.3.3) packages. Means of each dataset were calculated by randomly sampling 30 data points from each dataset 100 000 times and taking the mean of the resulting distribution. The Freedman‐Diaconis rule was chosen for histogram binning.^17^ Normality tests were performed on the data using the D’Agostino‐Pearson *K* ^2^ test.^18^ Where required, data were reshaped using a sigmoid function to enable the use of statistical tests that require normality.^19^ All statistical tests were two‐tailed and employed an alpha value of 0.05.

The mean is defined as *µ* and the standard deviation, *σ*. Where box‐plots are provided, whiskers illustrate 1.5 times the interquartile range and the black crosses represent outliers. Where 3D information is presented (i.e. a combination of translations or a combination of rotations), the distances are calculated as the modulus of the vector in the three Cartesian axes. Our analysis of confidence intervals was designed to encompass 95% of the sample population, this is calculated as *µ ± *2*σ*. Where asymmetric data are presented, the 95% Confidence Interval (CI) was calculated using the 2.5% to 97.5% percentiles.

## 3 RESULTS

Our analysis of patient positioning for intracranial SRS treatments is divided into three components: (a) patient setup, (b) patient intrafraction motion, and (c) the effects of treatment times on patient positioning. All of the data presented in this work have been de‐identified and provided in an SQL database as Supplementary material.

### 3.A Patient setup

**Fig. 1:**
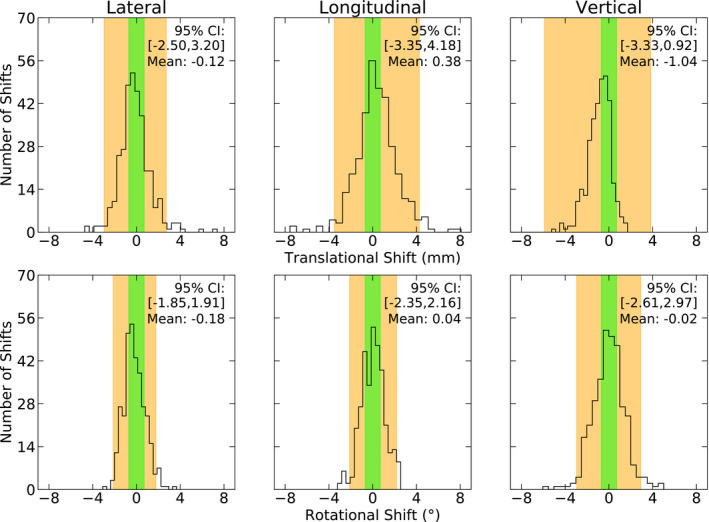
Patient setup accuracy using non‐ionising radiation methods are shown for n = 334, 6 DoF shifts. These values are calculated by the first x‐ray image set acquired with the ExacTrac image guidance system. The 0.7 mm, 0.7° action level is shown in green. The 95% Confidence Interval is shown in orange. Two vertical shifts of −12.50 mm and −39.24 mm are not shown in the corresponding histogram.

The initial setup of the patient uses no x‐ray imaging for alignment and is used to grossly align the patient. The first image set acquired with ExacTrac identifies the overall accuracy achieved during this setup process, the outcomes of which are shown in Fig. 1. These data include all initial setup shift data after patient resets have occurred, as they are also considered a new positioning procedure for the patient.

Normality tests showed that all of the axes were non‐normal with the exception of the longitudinal rotation axis (*P *<* *0.05). All translations and the lateral rotation contain either negative or positive asymmetry, which implies a bias exists towards either positive or negative shifts during patient setup. The largest bias is observed in the vertical translations whereby the mean value is −1.03 mm.

A Spearman’s Rank Correlation test was used to test for correlations between the six axes (*P *<* *0.05). Two combinations were weakly correlated (0.30 ≤ *r_s_ *< 0.50). The first of the two observed that as lateral rotations (pitch) become more positive, longitudinal translations become more negative (*r_s_* = −0.40). This observation could be attributed to the movement of the patient within the mask; as the patient rotates their head within the mask, small translations are introduced that also must be accounted for. The second correlation showed that as longitudinal rotations (roll) become more positive, vertical rotations (yaw) become more negative (*r_s_* = −0.46). The nature of this relationship is not obvious and is currently unable to be explained.

The largest observed shifts in the data were −39.24 mm and −6.04° in the vertical translations and rotations. Occasionally, large vertical shifts are introduced to help correct for pitch, which is limited to ±3°. In these cases, a large vertical translation is first applied followed by a smaller pitch correction. However, vertical rotations of this magnitude are typical of both couch and patient starting positions that are slightly rotated from the planned positions during setup. Given the asymmetry present in the data for each axis, we have chosen to present the 95% CI with 2.5% to 97.5% percentiles rather than standard deviations; however, for completeness, both are presented in Table 1. The 95% Confidence Intervals (CI), calculated by percentiles, range from −3.35 to 4.18 mm for translations and −2.61° to 2.97° for rotations.

**Table 1:** The 95% Confidence Intervals as calculated by the mean and standard deviation, and by the 2.5% to 97.5% percentiles are presented for comparison for Figs. 1, 2, 3.

Axis	Units	Initial setup				Initial verfification				Intrafaction			
		μ ± 2σ		2.5 to 97.5%		μ ± 2σ		2.5 to 97.5%		μ ± 2σ		2.5 to 97.5%	
Lateral translations	mm	−2.96	2.73	−2.50	3.20	−1.12	1.06	−0.82	0.79	−0.68	0.83	−0.65	0.80
Longitudinal translations	mm	−3.51	4.26	−3.35	4.18	−1.61	1.79	−0.67	2.11	−0.86	0.93	−0.83	0.93
Vertical translations	mm	−5.89	3.83	−3.33	0.92	−1.36	1.16	−1.58	0.80	−0.83	0.60	−0.76	0.53
Lateral rotations	*°*	−2.13	1.77	−1.85	1.91	−1.38	1.26	−1.92	0.57	−0.68	0.84	−0.67	0.75
Longitudinal rotations	*°*	−2.11	2.19	−2.35	2.16	−0.78	0.77	−0.68	0.52	−0.54	0.52	−0.57	0.55
Vertical rotations	*°*	−2.95	2.92	−2.61	2.97	−1.32	1.16	−2.27	0.63	−0.65	0.68	−0.67	0.71
Figure reference		Fig. 1				Fig. 2				Fig. 3			

**Fig. 2:**
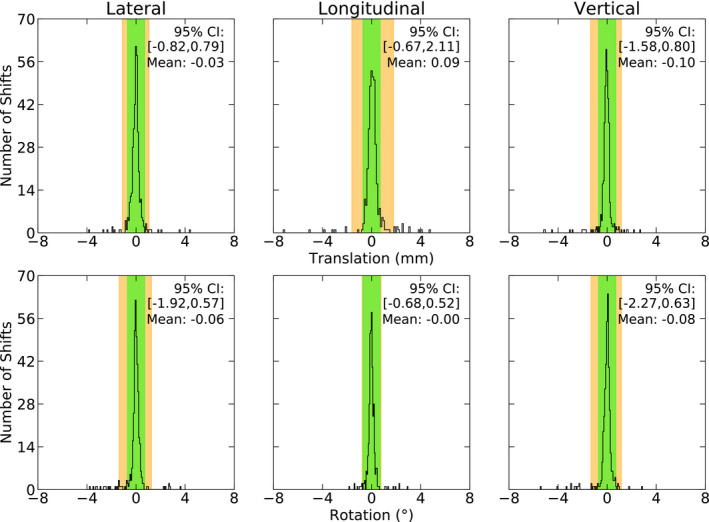
The residual patient setup (comprised of image guidance with ExacTrac only), is shown (*n* =376). This data includes all the recorded shifts that aim to bring the patient to within the 0.7 mm, 0.7° action level at the first planned couch angle. The 0.7 mm, 0.7° action level is shown in green. The 95% Confidence Interval is shown in orange.

The initial setup shifts in Fig. 1, as calculated by ExacTrac, were applied without verification and the patients were rotated to the first planned couch position. At the new couch position, using ExacTrac, the patient position was adjusted until all 6 DoF were within the action limit. The results of this verification process are shown in Fig. 2. Two cavity patients (12.5%) and six intact metastases patients (6.9%) required between one to two positioning resets over nine treatments during this setup phase, with a total of 11 resets.

Normal tests concluded that each of the six distributions in Fig. 2 are shown to be non‐normal, extremely narrow and highly skewed. The distributions are, however, centered within ±0.10 mm and ±0.08°. Again, a Spearman's Rank Correlation test was used to test for correlations between the six axes (*P *<* *0.05). Five correlations were observed in the data, however all of them were considered to be negligible (*r_s_ *<* *0.30), demonstrating inter‐axis independency. Thus, every shift is unique, and a shift observed in one axis does not come with the expectation of a companion shift in another axis.

Again, given the asymmetry present in the data, we have chosen to present the percentile ranges rather than standard deviations. The 95% CI’s range between −1.58 and 2.11 mm for translations and −2.27° and 0.63° for rotations, with the largest shifts recorded as −7.20 mm in the longitudinal and −5.54° in the vertical axes.

### 3.B Patient intrafraction shifts

**Fig. 3:**
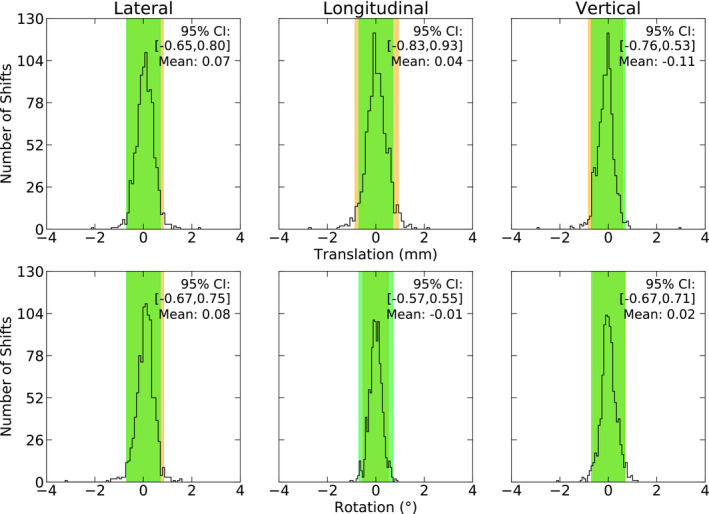
Patient intrafraction shifts as reported by ExacTrac are shown (*n* = 953). The green region represents the 0.7 mm, 0.7° threshold, and the orange region represents the 95% Confidence Interval.

The patient intrafraction shifts, as reported by ExacTrac, are shown in Fig. 3. Normality tests on each axis conclude that each axis is non‐normal, presenting with narrow distributions and minimal asymmetry. The mean of each axis is within ±0.11 mm and ±0.08°.

A Spearman’s Rank Correlation test was used to test for correlations between the six axes (*P *<* *0.05). Seven correlations were observed in the data, with all correlations considered negligible (*r_s_ *<* *0.30).

The 95% CI’s are sub‐millimeter, ranging from −0.83 to 0.93 mm and −0.67° to 0.75°. The largest translation observed was 3.00 mm in the vertical axis, and the largest rotation was −3.23° in the lateral axis (table roll). In this case, although the data are non‐normal, use of the standard deviation is appropriate due to the symmetry of the data; again, for consistency, both methods of calculating the 95% CI's have been included in Table 1.

Two intact metastases patients (2.3%) each required a single positioning reset, for a total of two resets during the intrafraction treatment phase. Both resets were performed at the end of the treatment session, prior to the last arc delivery.

**Fig. 4:**
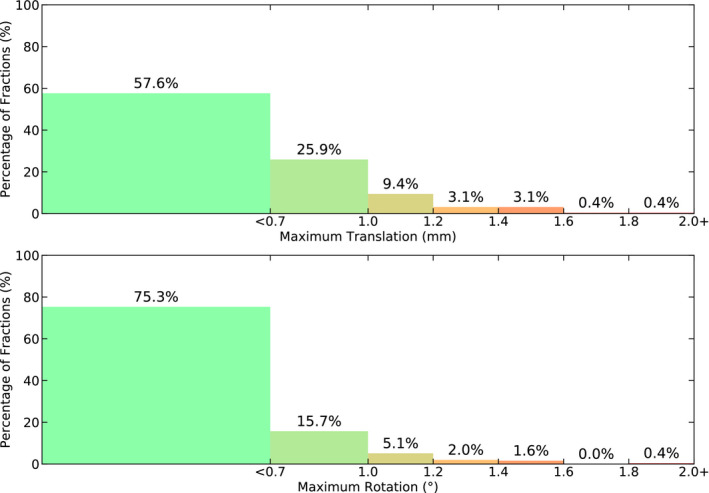
A differential histogram depicting the largest patient movement per treatment session is shown for (a) translations and (b) rotations separately.

The largest patient movements per treatment session are shown in Fig. 4. We observed that 42.4% of treatment fractions required a positioning correction greater than 0.7 mm and 0.7° during the treatment.

### 3.C Effect of couch angle

**Fig. 5:**
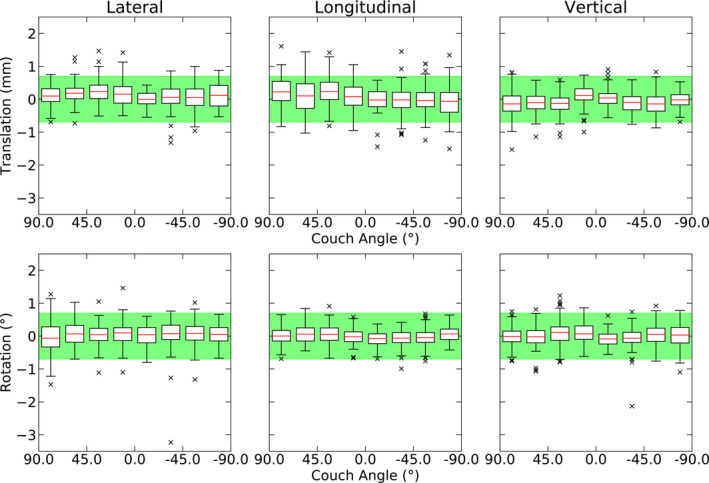
Patient intrafraction shifts as reported by ExacTrac are shown versus couch angle (*n* = 953). The green region represents the ±0.7 mm, ±0.7° threshold. Data is binned into 22.5° increments with sample sizes of 46 < *n* < 162.

Intrafraction shifts were split into 22.5° increments from C90 to C270 (90° to −90°) for each of the six axes. These intrafraction shifts are present as a function of couch angle in Fig. 5. A Spearman's Rank Correlation test was used to test for correlations between shifts in each of the axes and couch angle (*P *<* *0.05), however, no meaningful trends were observed. This again, demonstrates inter‐axis independency, where no one couch angle has the expectation of observing larger shifts compared to any other, highlighting the necessity of intrafraction imaging at every couch angle.

### 3.D Patient time on couch

**Fig. 6:**
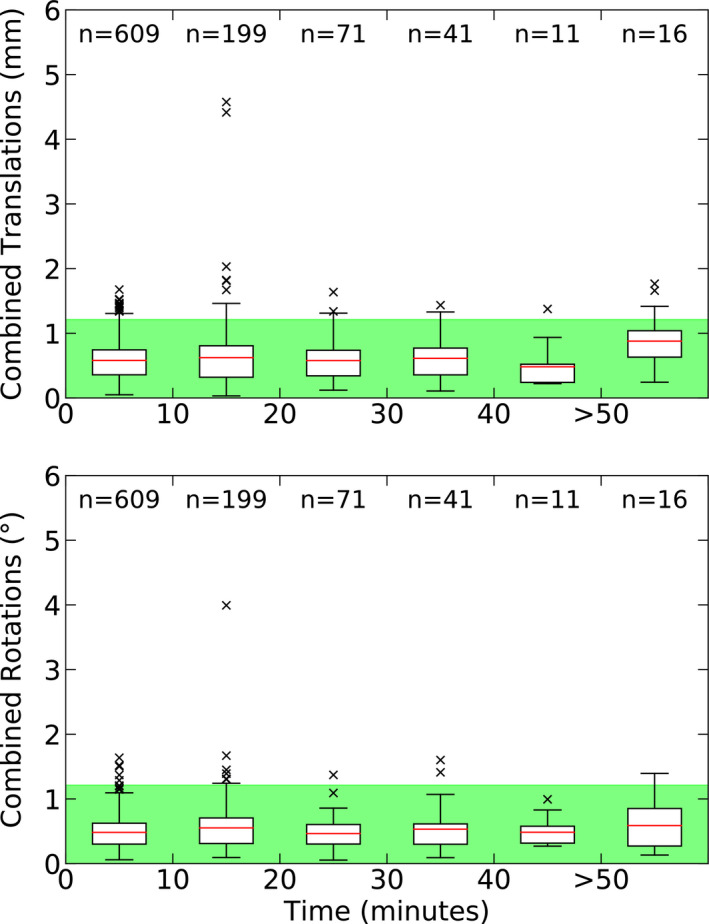
The magnitude of 3D translation and rotation vectors are binned into 10‐minute intervals. Zero time represents the beginning of patient treatment (the first image acquired by ExacTrac during patient setup). The maximum treatment time observed was 66 minutes.

Intrafraction patient shifts were explored with respect to time and are shown in Fig. 6. Patient treatment times ranged from 4 to 66 min (14 ± 11 min, *µ ± σ*). In this analysis, it was assumed that patients were never removed from the linac couch when multiple isocenters were treated in the same treatment session. As such, the data presented include only the intrafraction patient shifts for the entire treatment session. Time points are determined as the passing of time since the first x‐ray acquisition with ExacTrac, not from the time the patient first laid on the linac couch.

A Spearman's Rank Correlation test was used to test for correlations between each of the six axes and time (*P *<* *0.05). A correlation between rotations and all time points was observed, although was considered to be negligible (*r_s_ *<* *0.30). A similar negligible correlation was also observed for treatment times below the mean treatment duration (<14 min), but not for shifts that occurred at time points ≥ 14 min. No correlation was found between translations and treatment duration. A meaningful correlation, however, was observed between the number of isocenters and treatment duration; longer treatment times were associated with the treatment of multiple isocenters (*r_s_* = 0.67), which is expected.

In all of the data shown, there were three treatments (1.16%) where a patient did not require any adjustments to their position; this is inclusive of the initial setup, verification and intrafraction portions of the treatment.

### 3.E Categorical comparisons

The intrafraction data presented in Fig. 3 were divided into single‐ versus multi‐isocenter patients and SRS versus SRT patients to identify any statistical differences between the cohorts. The data were reshaped by a sigmoid function (tan^−1^ *x*) for use in a Welch’s unequal variances t‐test. A statistically significant difference was observed between the vertical translations and longitudinal rotations for the single‐ and multi‐isocenter patients, where the means of each dataset were 0.04 mm and 0.07° respectively. For the SRS versus SRT patients, a significant difference was found between the longitudinal and vertical translations, although the difference in means was again, small (<0.08 mm).

## 4 DISCUSSION

The initial patient setup, exclusive of x‐ray imaging (Fig. 1), was shown to vary by a large amount between the six axes. The off‐centered nature of the longitudinal and vertical translation axes indicate a systematic positioning offset of about 0.4 to 1 mm of the patient anatomy to the IR mask frame; this offset originates during application of the mask and setup in treatment position using both manual and IR positioning. These setup data are not shown in other reports. The closest comparison that can be made is to the work of Zhang et al.,^10^ who provide both initial setup and verification alignment data for patient setup. In their study, initial alignment (without x‐rays) is performed using the in‐room lasers, not an IR system, for which the means of our data are considerably smaller. The rotational shifts are exceptionally large in Zhang et al., however, they state that rotational errors were ignored as correcting for them did not provide any clinical benefit.

From our review, our setup verification data (shown in Fig. 2) are in agreement with all other studies, which observe verification means of about ±0.1 mm, ±0.1°. After the verification shifts were applied, the spread of shift data in all six axes was greatly reduced and became centered about zero and the 95 % CI’s also approached the desired 0.7 mm, 0.7° action level. The largest observed shift reduced from 39.24 mm and 6.04° in the initial setup (no image guidance) to 7.20 mm and 5.54° in the first arc alignment phase (image guidance with verification). Given that the maximal shifts do not reduce largely between the initial and verification alignments, we can conclude that applying one correctional shift is not always enough to completely correct the patient position, instead multiple alignments are often required.

The reduction in patient position variation after the initial x‐ray acquisition and verification shifts reinforces the necessity for the use of x‐ray based pretreatment patient setup in SRS/SRT. Further, this also highlights that although the IR system achieves alignment to within 0.7 mm, that is only for the mask frame. The patient position within the mask can only be corrected with imaging that is capable of correcting based on internal patient anatomy. Only after the patient position is verified, can the IR system indicate potential patient shifts throughout treatment, as promoted by Spadea et al.^8^ Although, we propose that IR monitoring of the mask has significant limitations in situations where single isocenters are employed for multiple metastases, as the IR system only registers the patient position in 3 DoF and will not account for any rotation discrepancies.

The patient intrafraction motion data, presented in Figs. 3, 4, 5, 6, depict stable patient motion throughout treatment. Figure 3 shows that 95 % of patients will move less than 0.93 mm and 0.75° during their treatment for any given axis, while Fig. 4 shows that the largest intrafraction movement for 95 % of patients will be within 1.2 mm, 1.2°. However, as shown in Fig. 4, 42.4 % of fractions required a shift larger than our institutional tolerance for correction (0.7 mm, 0.7°). Therefore, without intrafraction imaging or monitoring, the observed patient movements would go uncorrected and our CTV‐PTV expansion margins of 1 mm (intact metastases cohort) and 2 mm (cavity cohort) would no longer be suitable, despite use of a full‐face SRS‐specific thermoplastic mask. Inter‐axis independence was also demonstrated for patient intrafraction motion. Independence between axes infers that corrections are required more often as a small or large correction in one axis does not coincide with a similar correction in another axis.

Of the studies who provided 6 DoF intrafraction motion data, the magnitude and spread of our data is only in agreement with that reported by Lewis et al.^15^ Although not directly comparable, our patient intrafraction shift data are also smaller than that reported by Guckenberger et al. (2012), Badakhshi et al. (2013) and Tarnavski et al. (2016).^7^, ^12^, ^14^ It should be noted that Guckenberger et al. (2012) employed cone beam CT for image guidance and simulated intrafraction motion by using pre‐ and post‐treatment imaging. However, Badakhshi et al. (2013), Tarnavski et al. (2016) and Lewis et al. (2018) each used ExacTrac with a 6 DoF couch with varying time periods between imaging. This study provides the most granular intrafraction motion data all the aforementioned studies.

Each study in our literature review of both initial and intrafraction patient shifts presented data only by mean values and various multiples of the standard deviation. It is quite common that the data are neither symmetrically distributed nor statistically normal, and as such, it is not always appropriate to present the confidence intervals by using this method.^19^ In our study we have provided 95 % CIs calculated with two methods, using the mean and standard deviation and by using percentiles. The initial setup and verification data presented in Figs. 1 and 2 highlight the limitation of using 95% CIs that are calculated by using the standard deviation to represent the data alone. For example, from Table 1, the longitudinal translations presented for the initial verification shifts (Fig. 2) show that using *μ* ± 2*σ* to calculate the 95% CI results in a range of (−1.61, 1.79) mm while using percentiles gives (−0.67, 2.11) mm. By considering only the confidence interval as calculated by the mean and standard deviation, near symmetrical patient motion would be expected, where in fact the confidence interval does not reflect the actual patient data. If, instead, percentiles were used to calculate the confidence interval, it would be apparent that patients are systematically more superiorly positioned by about 1 mm. By using only the standard deviation method for calculating confidence intervals, incorrect conclusions about the data will be drawn. Using percentiles to calculate confidence intervals will highlight any asymmetries in the data (as shown in our data) and the effects of outliers, which will be otherwise missed. Both methods should be used in future studies as it is imperative that the data are well understood before being used to impact decisions such as margin calculations or choosing positioning action levels. Additionally, it also makes the data easier to compare against other studies.

Schmidhalter et al. (2014) observed very strong sinusoidal trends attributable to couch walk‐out in their dataset^11^; however, in our dataset, we do not observe such trends so strongly. The weakness of our identified trends implies that couch walk‐out on our specific linac is minimal and adequately accounted for during QA. This finding is important as imaging is often restricted once the couch is rotated, which is especially problematic for on‐board imaging systems.

In situations where mask material is removed in order to facilitate other means of image guidance (such as optical systems), patient positioning results have been shown to be comparable to that of ExacTrac.^20^ Schmidhalter et al. also attributed some positioning inaccuracies to patient weight loss and the subsequent increased movement within the mask, although in our study this is not an issue as we are not treating more than 5 fractions.^11^ Occasionally, patient swelling can also occur. In such a situation, an extra “spacer” is placed between the two halves of the patient mask to reduce the pressure on the patient’s face. However, given that the swelling reduces the amount of space within the mask, we do not expect that it has a detrimental impact on patient positioning. In severe cases of swelling, the mask may be remade, or more rarely, parts of the mask may be cut away to relieve the pressure. However, given that cutting sections of a mask away for optical surface tracking techniques has shown to provide similar alignment results to that of ExacTrac,^20^ we do not expect that such a modification (as rare as it is) would grossly impact our results.

Regarding patient motion with respect to treatment duration, Hoogeman et al. (2008) used stereoscopic imaging to study uncorrected patient displacement from isocenter and found significant correlations between patient intrafraction motion and time.^13^ Guckenberger et al. (2012) used positioning outcomes after corrected patient displacement and also found that intrafraction positional errors were significantly correlated with time.^7^ Tarnavski et al. (2016), in an ExacTrac‐based study, also found that patient movement (greater than 2 mm or 2°) increased with treatment durations of >10 min.^14^ However, their observed movements are much larger than what is observed in our study, which would suggest a difference in the immobilization systems used. In their study, two different mask systems were used (a Civco Posicast and Brainlab Thermoplastic mask) and were grouped together for their analysis, however, in our study, the Klarity mask was used. There may be differences in immobilization and comfort between different masks which may result in variations in intrafraction motion.

Conversely, in our data, a negligible correlation was observed between rotations and all treatment durations, as well as for shifts below the mean treatment duration of 14 min. Such weak correlations do not appear to be meaningful as all patient motion is well within the magnitude of the (3D) 0.7 mm tolerance. From our data, it is expected that patients will exhibit the same movement behavior throughout their entire treatment for treatment durations of <60 min.

Lewis et al. (2018) provided both initial patient setup and patient intrafraction motion data that are smaller than what is observed in this study.^15^ Our data are not directly comparable as they do not provide 6 DoF information nor do they image as frequently. However, we are in agreement with the findings that patient movement throughout treatment is limited by the thermoplastic mask (with similar maximal translations being observed throughout treatment) and that there is no correlation between patient motion and treatment duration.

A large caveat of any patient intrafraction motion studies is that the results are intimately tied to the stability of the immobilization system and may also be affected by operator skill and experience (or changes in patient anatomy as previously discussed). Ohtakara et al. (2012) explored differences between various thermoplastic mask systems and found that patient positioning outcomes is expected to change between masks,^16^ whereas Guckenberger et al. (2012) found no difference between single and double layer masks.^7^ Thus, in the future studies or application of these results to any clinical use would require careful consideration of how the immobilization apparatus affects patient movement.

Further to this, several statistically significant correlations were observed in our data between patient motion in each axis and tumor displacement. Although all trends were deemed negligible, they could provide a good model for the design and recommendations in future mask immobilization systems.

In our study, patients were planned with either DCAT or IMRT and one isocenter per target which led to, in some cases, treatment durations of up to an hour. However, in recent years, mono‐isocenter multi‐target VMAT is becoming a popular approach for SRS.^21^ Mono‐isocenter VMAT techniques offer large reductions in treatment times, however, they also come with challenges in patient positioning. Using the real patient data provided in this study, and several existing studies on the effects of patient position in mono‐isocenter techniques,^22^, ^23^ margins or action levels for image guidance could be inferred.

This work provides a statistically sound overview of patient setup and intrafraction motion throughout SRS and SRT treatments. The raw data provided and review of our intrafraction data allows for sampling it in future studies where real patient data are required in order to study dosimetric effects of other treatment techniques such as single isocenter, multi‐metastases treatments, complete more in‐depth studies on the data or provide a time point in which future data can be adequately compared.

## 5 CONCLUSION

We have retrospectively reviewed the variation in our patient setup and intrafraction shifts for intracranial SRS treatments. We have shown out of 101 patients and 258 fractions, 42.4% of treatments required a positioning correction greater than 0.7 mm, 0.7° during treatment. We found that the immobilization mask limited patient movement to within approximately 3 mm, 3°. Patient position, however, must still be corrected, but did not increase with time or couch rotation. With our findings, we conclude that intrafraction 6 DoF patient position monitoring is essential for cranial SRS treatments to enable typically used 1–2 mm CTV‐PTV margins.

## CONFLICT OF INTEREST

Tomas Kron and Nicholas Hardcastle receive funding from Varian Medical Systems for an unrelated project.

## Supporting information


**Data S1.** An anonymised SQL database containing all patient shift data stored by ExacTrac for all treatments investigated in this work.
